# Operation of Tracheotomy in Croup

**Published:** 1856-04

**Authors:** E. J. Roach

**Affiliations:** Atlanta, Georgia


### ARTICLE IV.

*Operation of Tracheotomy in Croup.* By E. J. Roach, M. D., of Atlanta, Georgia.

The discrepancy of opinion among the Medical Profession, in regard to this operation, together with the frequent occurrence of croup and its fatality, must be my apology for the above caption.

Croup, or Cynanche Trachealis of Cullin, as generally considered, is expressive of an inflammatory condition of the larynx or trachea, characterized by spasms of the larynx, and stridulous breathing; the inflammation terminating, in the majority of cases, in the exudation of false membrane. Croup is essentially a disease of early life, and common in all countries; but is much influenced by peculiarities of climate and locality. It is not exclusively confined to Northern regions, but prevails, on the contrary, sometimes, to an alarming extent, in the South. It is endemic in some districts; low countries, wet soil, and a damp atmosphere, are pre-disposing causes of croup. A proof of this is its rare occurrence in cities and towns, and its frequency in rural districts.

Croup is classified into three different forms. Of the pseudomembranous I propose to speak only, as this is the one in which the operation is generally performed; and there is no disease to which childhood is subject, and which the members of the Medical Profession are called upon to treat, that involves more discretion in treatment, and excites their sympathy more, tor the little sufferer, than this. It sometimes baffles all remedial agents, and in many instances, we are compelled to witness its last gasp for life, amidst the agonies and tears of an anxious mother.

The symptoms of this form of croup are so familiar to every physician, that it is not necessary for me to speak of them in detail. The extinction of the voice, suppressing of the cough, the change from stridulous to sibilant breathing, and increase difficulty of respiration, all prove the patient to be in articulo mortis. It is in cases of these symptoms, after all other remediable means have failed, that the operation of Tracheotomy is recommended and performed.

Tracheotomy was first advocated on theoretical principles, by Dr. Home. But, however, its utility was not put to the test for several years afterwards. I believe the first instance we have of its success, was by M. Bretanneau, in 1825; since then, it has been performed, successfully, in a number of cases, by French, English, and American surgeons, but with a great deal more success with the former. The English and American, with whom it has been so unfavorable, scarçely look on it as a justifiable operation, relying, generally, on the other remedies in common use. It is argued, by some, that the per cent. in which the operation is successful, is owing to its performance in the early stages of the disease, and before other means have been persevered in, or when there was no need for it; likewise, that in many instances, it hastens the death of the sufferer, by augmenting the bronchial disturbance, which is always more or less embarrassing in Croup. It is also alleged that the greater portion of the aperture of the larynx has been found free in fatal cases of Croup. These are some of the most important arguments against the operation, while on the contrary, it is argued by those favoring the operation, and particularly the French surgeons, that this is not generally the case; but that the increased bronchial disturbance is more frequently owing, says Trousseau, to the ineffectual opening in the Trachea, and that the operation is not performed merely for removing from the windpipe the membrane which prevented the ingress of air into the lungs, but that it is done to relieve the spasmodic contractions which the inflammation occasions. Each of these organs seem to admit of plausibility, particularly the former, when we have in mind that fatal cases rarely occur, without the bronchial tubes are seriously affected. Admitting then, that in many instances, the operation increases the bronchial disturbance, we cannot but disapprove of it. On the contrary, the latter is doubtful, as the ratio of success, in the cases operated on by Trousseau himself, will not make it a justifiible proceeding, taking into consideration the favorable condition of many of the patients operated on.

Perhaps, already, I have said too much upon this subject, but yet I thought it might not be improper to state the circumstances which generally make the Profession hesitate in expressing a decided opinion against Tracheotomy, as well as those in favor, before expressing my own unqualified disapprobation of the operation in cases of Croup.

